# Journey to self‐determination in Indigenous cognitive health research in Canada

**DOI:** 10.1002/alz70855_099346

**Published:** 2026-01-05

**Authors:** Jennifer D Walker, Pamela Roach

**Affiliations:** ^1^ McMaster University, Hamilton, ON, Canada; ^2^ University of Calgary, Calgary, AB, Canada

## Abstract

**Background:**

Indigenous communities in Canada call for decolonized dementia research, given their higher dementia rates and risk. Over the past 10 years, CCNA has supported increasing levels of self‐determination in Indigenous dementia research.

**Method:**

Initially, Indigenous research was prioritized as one half of a funded Team that supported First Nations‐focused research. In CCNA's second phase, we strengthened self‐determined Indigenous‐led research through a dedicated Indigenous‐led research Team and through the establishment of the Indigenous Cognitive Health Program, a cross‐cutting initiative designed to promote learning across the whole CCNA network. CCNA made deliberate efforts to build strength and support Indigenous cognitive health researchers, trainees, community partnerships, and community‐based research advisory structures. The Indigenous team worked together to envision the next stage of Indigenous self‐determination and decolonization of Indigenous dementia research through two in‐person and two online gatherings in 2023 and 2024.

**Result:**

Two influential research outputs were the Canadian Indigenous Cognitive Assessment and the assessment of the Brain Health Pro platform. Since 2019, our Indigenous scientific team has added 5 Indigenous researchers, submitted 8 new funding proposals, hosted 15 webinars on Indigenous cognitive health research, and launched a website to support pathways to culturally safe Indigenous health research approaches.

**Conclusion:**

These efforts have contributed to a substantial shift in the readiness of Indigenous communities in Canada to address rising numbers of people living with dementia. The future vision is for an Indigenous self‐determined Community‐Centred Indigenous Cognitive Health Network (CICHN) in Canada that will build on the past 10 years of increasing capacity.

